# Supplementary material for: Microbial Community and Abundance of Selected Antimicrobial Resistance Genes in Poultry Litter from Conventional and Antibiotic-Free Farms
Source: Antibiotics (Basel). 2023 Sep 19;12(9):1461. doi: 10.3390/antibiotics12091461 (PMC10525487; doi:10.3390/antibiotics12091461)
Supplement: Supplementary file 1 [file antibiotics-12-01461-s001.zip › Supplementary files/Table S1.docx]

**Supplementary Table 1.** The Spearman correlation coefficient (r) and significant p value (p) (p<0.05) of bacterial families identified in this study.

| **Bacterial Family** |  | **r** | **p** |
| --- | --- | --- | --- |
| Dermabacteraceae | Corynebacteriaceae | 0.7932 | 0.0099 |
| Brevibacteriaceae | Corynebacteriaceae | 0.8358 | 0.0010 |
| Staphylococcaceae | Corynebacteriaceae | 0.8841 | 0.0000 |
| Lachnospiraceae | Ruminococcaceae | 0.8834 | 0.0000 |
| Oscillospiraceae | Ruminococcaceae | 0.9311 | 0.0000 |
| Lachnospiraceae | Bacteroidaceae | 0.7945 | 0.0093 |
| Acidaminococcaceae | Campylobacteraceae | 0.8705 | 0.0001 |
| Clostridia_UCG014 | Ruminococcaceae | 0.9167 | 0.0000 |
| Clostridia_UCG014 | Bacteroidaceae | 0.8109 | 0.0041 |
| Rhodospirillalesuncultured | Campylobacteraceae | 0.8595 | 0.0002 |
| BacilliRF39f__RF39 | Ruminococcaceae | 0.7983 | 0.0077 |
| Erysipelotrichaceae | Ruminococcaceae | 0.7957 | 0.0088 |
| Erysipelotrichaceae | Bacteroidaceae | 0.7631 | 0.0381 |
| Oscillospiraceae | Lachnospiraceae | 0.8421 | 0.0007 |
| Brevibacteriaceae | Dermabacteraceae | 0.9254 | 0.0000 |
| Carnobacteriaceae | Sphingobacteriaceae | 0.9078 | 0.0000 |
| Staphylococcaceae | Brevibacteriaceae | 0.7609 | 0.0416 |
| Erysipelotrichaceae | Lachnospiraceae | 0.7600 | 0.0431 |
| Clostridia_vadinBB60_group | Oscillospiraceae | 0.7578 | 0.0472 |
| Clostridia_UCG014 | Lachnospiraceae | 0.8185 | 0.0027 |
| BacilliRF39f__RF39 | Lachnospiraceae | 0.8401 | 0.0007 |
| Clostridia_UCG014 | Oscillospiraceae | 0.9256 | 0.0000 |
| Rhodospirillalesuncultured | Acidaminococcaceae | 0.7725 | 0.0256 |
| Butyricicoccaceae | Oscillospiraceae | 0.8064 | 0.0051 |
| Erysipelotrichaceae | Lachnospiraceae | 0.7610 | 0.0415 |
| Erysipelotrichaceae | Oscillospiraceae | 0.8251 | 0.0018 |
| Moraxellaceae | Sphingobacteriaceae | 0.8073 | 0.0049 |
| Moraxellaceae | Alcaligenaceae | 0.8782 | 0.0000 |
| Dietziaceae | Sphingobacteriaceae | 0.9142 | 0.0000 |
| Christensenellaceae | Oscillospiraceae | 0.7710 | 0.0273 |
| Rhodospirillalesuncultured | Pasteurellaceae | 0.8343 | 0.0011 |
| Dietziaceae | Carnobacteriaceae | 0.8283 | 0.0015 |
| Tannerellaceae | Barnesiellaceae | 0.8519 | 0.0003 |
| Desulfovibrionaceae | Pasteurellaceae | 0.7980 | 0.0078 |
| Rhodospirillalesuncultured | Rikenellaceae | 0.7626 | 0.0102 |
| Clostridia_vadinBB60_group | Erysipelatoclostridiaceae | 0.8301 | 0.0014 |
| BacilliRF39f__RF39 | Erysipelatoclostridiaceae | 0.8459 | 0.0005 |
| Acholeplasmataceae | Erysipelatoclostridiaceae | 0.8214 | 0.0023 |
| Erysipelotrichaceae | Erysipelatoclostridiaceae | 0.7926 | 0.0389 |
| Butyricicoccaceae | Clostridia_vadinBB60_group | 0.7727 | 0.0253 |
| BacilliRF39f__RF39 | Clostridia_vadinBB60_group | 0.7701 | 0.0284 |
| Acholeplasmataceae | Clostridia_vadinBB60_group | 0.9845 | 0.0000 |
| Erysipelotrichaceae | Clostridia_vadinBB60_group | 0.8582 | 0.0002 |
| Eubacterium_coprostanoligenes_gr | Clostridia_vadinBB60_group | 0.7597 | 0.0437 |
| Rhodospirillales uncultured | UCG010 | 0.8343 | 0.8343 |
| Pasteurellaceae | Marinifilaceae | 1 | 0.0000 |
| Pasteurellaceae | UCG010 | 1 | 0.0000 |
| Tannerellaceae | Clostridia_vadinBB60_group | 0.8221 | 0.0022 |
| Victivallaceae | Pasteurellaceae | 0.9962 | 0.0000 |
| Clostridium_methylpentosum_group | Pasteurellaceae | 0.9962 | 0.0000 |
| Victivallaceae | Rhodospirillalesuncultured | 0.8311 | 0.0013 |
| Clostridium_methylpentosum_group | Rhodospirillalesuncultured | 0.8311 | 0.0013 |
| Izemoplasmatales | Pasteurellaceae | 0.9962 | 0.0000 |
| Defluviitaleaceae | Pasteurellaceae | 0.9962 | 0.0000 |
| Izemoplasmatales | Rhodospirillalesuncultured | 0.8311 | 0.8311 |
| Defluviitaleaceae | Rhodospirillalesuncultured | 0.8341 | 0.0011 |
| Erysipelotrichaceae | Clostridia_UCG014 | 0.8114 | 0.0039 |
| Acholeplasmataceae | Butyricicoccaceae | 0.7674 | 0.0318 |
| Erysipelotrichaceae | Butyricicoccaceae | 0.8401 | 0.0007 |
| Christensenellaceae | Butyricicoccaceae | 0.7824 | 0.0164 |
| Dietziaceae | Flavobacteriaceae | 0.7569 | 0.049 |
| Marinifilaceae | Desulfovibrionaceae | 0.7980 | 0.0078 |
| Erysipelotrichaceae | Desulfovibrionaceae | 0.7980 | 0.0078 |
| Acholeplasmataceae | Erysipelotrichaceae | 0.8414 | 0.0007 |
| Acholeplasmataceae | Tannerellaceae | 0.8287 | 0.0015 |
| UCG010 | Marinifilaceae | 1 | 0.0000 |
| Marinifilaceae | Victivallaceae | 0.9962 | 0.0000 |
| Marinifilaceae | Clostridium_methylpentosum_group | 0.9962 | 0.0000 |
| Erysipelotrichaceae | Tannerellaceae | 0.7735 | 0.0245 |
| Erysipelotrichaceae | Christensenellaceae | 0.7677 | 0.0313 |
| Peptostreptococcales-Tissierellales | Moraxellaceae | 0.8311 | 0.0013 |
| Eubacterium_coprostanoligenes_gr | Christensenellaceae | 0.8584 | 0.0002 |
| Eubacterium_coprostanoligenes_gr | Eggerthellaceae | 0.8229 | 0.0021 |
| Christensenellaceae | Eggerthellaceae | 0.7754 | 0.0226 |
| Sphingobacteriaceae | Peptostreptococcales-Tissierellales | 0.7575 | 0.0477 |
| Sphingobacteriaceae | Anaerovoracaceae | 0.8120 | 0.0038 |
| Sphingobacteriaceae | Eggerthellaceae | 0.8310 | 0.0013 |
| Victivallaceae | Izemoplasmatales | 1 | 0.0000 |
| Victivallaceae | Defluviitaleace | 0.9962 | 0.0000 |
| Sutterellaceae | Victivallaceae | 1 | 0.0000 |
| Defluviitaleaceae | Clostridium_methylpentosum_group | 0.9962 | 0.0000 |
| Izemoplasmatales | Clostridium_methylpentosum_group | 1 | 0.0000 |
| Izemoplasmatales | Defluviitaleace | 0.9962 | 0.0000 |
