# Supplementary figures and images for: Microbial Community and Abundance of Selected Antimicrobial Resistance Genes in Poultry Litter from Conventional and Antibiotic-Free Farms
Source: Antibiotics (Basel). 2023 Sep 19;12(9):1461. doi: 10.3390/antibiotics12091461 (PMC10525487; doi:10.3390/antibiotics12091461)

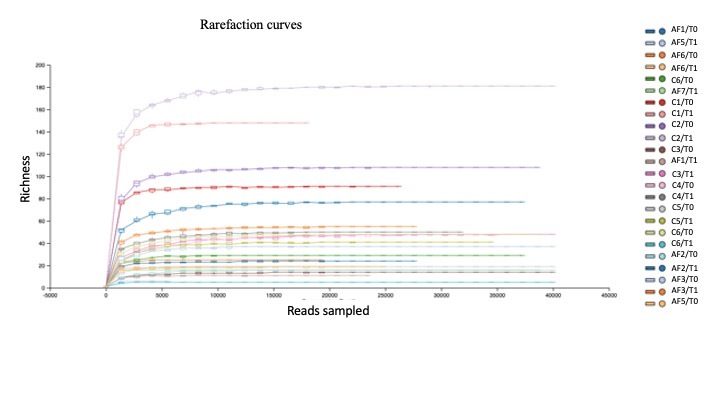

Supplement: Supplementary file 1 [file antibiotics-12-01461-s001.zip › Supplementary files/Figure S1.jpg]
